# Self-modulation of motor cortex activity after stroke: a randomized controlled trial

**DOI:** 10.1093/brain/awac239

**Published:** 2022-08-12

**Authors:** Zeena-Britt Sanders, Melanie K Fleming, Tom Smejka, Marilien C Marzolla, Catharina Zich, Sebastian W Rieger, Michael Lührs, Rainer Goebel, Cassandra Sampaio-Baptista, Heidi Johansen-Berg

**Affiliations:** Wellcome Centre for Integrative Neuroimaging, University of Oxford, Oxford OX3 9DU, UK; Wellcome Centre for Integrative Neuroimaging, University of Oxford, Oxford OX3 9DU, UK; Wellcome Centre for Integrative Neuroimaging, University of Oxford, Oxford OX3 9DU, UK; Wellcome Centre for Integrative Neuroimaging, University of Oxford, Oxford OX3 9DU, UK; Wellcome Centre for Integrative Neuroimaging, University of Oxford, Oxford OX3 9DU, UK; Wellcome Centre for Integrative Neuroimaging, University of Oxford, Oxford OX3 9DU, UK; Department of Cognitive Neuroscience, Maastricht University, 6229 EV Maastricht, The Netherlands; Research Department, Brain Innovation B.V., 6229 EV Maastricht, The Netherlands; Department of Cognitive Neuroscience, Maastricht University, 6229 EV Maastricht, The Netherlands; Research Department, Brain Innovation B.V., 6229 EV Maastricht, The Netherlands; Wellcome Centre for Integrative Neuroimaging, University of Oxford, Oxford OX3 9DU, UK; Institute of Neuroscience and Psychology, University of Glasgow, Glasgow G61 1QH, UK; Wellcome Centre for Integrative Neuroimaging, University of Oxford, Oxford OX3 9DU, UK

**Keywords:** stroke, real-time fMRI, neurofeedback, motor cortex, white matter

## Abstract

Real-time functional MRI neurofeedback allows individuals to self-modulate their ongoing brain activity. This may be a useful tool in clinical disorders that are associated with altered brain activity patterns. Motor impairment after stroke has previously been associated with decreased laterality of motor cortex activity. Here we examined whether chronic stroke survivors were able to use real-time fMRI neurofeedback to increase laterality of motor cortex activity and assessed effects on motor performance and on brain structure and function.

We carried out a randomized, double-blind, sham-controlled trial (ClinicalTrials.gov: NCT03775915) in which 24 chronic stroke survivors with mild to moderate upper limb impairment experienced three training days of either Real (*n* = 12) or Sham (*n* = 12) neurofeedback. Assessments of brain structure, brain function and measures of upper-limb function were carried out before and 1 week after neurofeedback training. Additionally, measures of upper-limb function were repeated 1 month after neurofeedback training. Primary outcome measures were (i) changes in lateralization of motor cortex activity during movements of the stroke-affected hand throughout neurofeedback training days; and (ii) changes in motor performance of the affected limb on the Jebsen Taylor Test (JTT).

Stroke survivors were able to use Real neurofeedback to increase laterality of motor cortex activity within (*P* = 0.019), but not across, training days. There was no group effect on the primary behavioural outcome measure, which was average JTT performance across all subtasks (*P* = 0.116). Secondary analysis found improvements in the performance of the gross motor subtasks of the JTT in the Real neurofeedback group compared to Sham (*P* = 0.010). However, there were no improvements on the Action Research Arm Test or the Upper Extremity Fugl–Meyer score (both *P* > 0.5). Additionally, decreased white-matter asymmetry of the corticospinal tracts was detected 1 week after neurofeedback training (*P* = 0.008), indicating that the tracts become more similar with Real neurofeedback. Changes in the affected corticospinal tract were positively correlated with participants neurofeedback performance (*P* = 0.002).

Therefore, here we demonstrate that chronic stroke survivors are able to use functional MRI neurofeedback to self-modulate motor cortex activity in comparison to a Sham control, and that training is associated with improvements in gross hand motor performance and with white matter structural changes.

## Teaser

In a randomized sham-controlled trial, Sanders *et al.* show that chronic stroke survivors with hemiplegia are able to use real-time functional MRI neurofeedback to self-modulate motor cortex activity, resulting in improvements in gross motor performance of the affected limb and structural brain changes.

## Introduction

Stroke survivors often suffer debilitating long-term motor impairments of the upper limb, which can have a negative impact on activities of daily living^1^ and quality of life.^2^ Rehabilitation typically focuses on the stroke-affected limb; however, there has been increased interest in interventions that target the brain (e.g. brain stimulation), in the hope of boosting rehabilitation effects and further improving motor function. Lateralization of motor cortex activity has been found to be reduced in stroke survivors when moving their stroke-affected limb.^3^ This reduction in lateralization has previously been associated with worse motor outcomes^4^; however, contralesional activity has also been suggested to play a compensatory role in some stroke survivors, particularly those with greater damage to the corticospinal tract.^5^ Previous brain stimulation studies typically target motor cortex activity and many attempt to promote lateralization of activity towards the stroke-affected motor cortex.^6,7^

A novel alternative approach, which harnesses the brains intrinsic capacity for activity modulation, is for stroke survivors to learn to self-modulate brain activity through real-time functional MRI (fMRI) neurofeedback (NF). Previous work has shown some promising behavioural effects of fMRI NF,^8–10^ and fMRI NF has been explored in various clinical conditions associated with aberrant brain activity patterns, for example depression,^11,12^ pain disorders^13,14^ and phobias.^15,16^ Functional MRI NF has also been suggested as a potentially useful tool in stroke rehabilitation^17,18^; however, to date no randomized, sham-controlled trials have been published. Previous pilot studies have shown that stroke survivors appear to be able to use fMRI NF to control activity in a variety of motor regions,^19–21^ demonstrating feasibility of this approach. After sufficient training, participants receiving NF training may be able to maintain the ability to self-modulate brain activity outside of the training sessions, leading to potentially long-lasting improvements (for review see Thibault *et al*.^22^).

Here we carried out a registered double-blind, sham-controlled trial to investigate the efficacy of three sessions of real-time fMRI NF in chronic stroke survivors with ongoing upper-limb impairments. The primary outcome measures of this trial were changes in ‘lateralization of motor cortex activity' during movements of the stroke-affected hand and changes in *motor performance* as assessed using the Jebsen Taylor Test (JTT).^23^ Additionally, secondary outcomes included measures of short- and long-term NF learning transfer, where participants’ ability to maintain changes in brain activity was assessed, as well as changes in clinical scores and brain structure.

## Materials and methods

### Study design

A double-blind, randomized controlled trial (ClinicalTrials.gov: NCT03775915) was carried out to examine chronic stroke survivors’ ability to use real-time fMRI NF to increase laterality of motor cortex (M1) activity during affected hand movements. Effects of NF on motor performance were assessed using the JTT as well as clinical measures. Additionally effects on brain structure were assessed using diffusion-weighted imaging (DWI), and effects on brain function during a visuomotor squeeze task were assessed using task-based fMRI and EEG. A parallel design was selected to avoid carryover effects, as it is currently unclear how long NF effects last.^24^

One-hundred and seventy-three chronic (>6 months post-stroke) stroke survivors were contacted to take part in the study between January 2018 and March 2020. Of these, 115 were screened to assess whether they met the inclusion criteria (see recruitment flow chart Supplementary Fig. 1). Initial inclusion criteria were: aged 18–85 years, prior symptomatic stroke with ongoing effects on upper limb movements on one side of the body and some residual movement in the stroke-affected hand. Exclusion criteria included MRI scanning contraindications, previous history of neurological or psychiatric illness and limited communication or inadequate understanding of instructions. In June 2018 criteria were expanded to allow more participants to be included: the upper age limit was removed and participants with more than one stroke could be included provided only one side of the body was affected. The trial was terminated in March 2020 due to the COVID-19 lockdown before the full sample (30 participants) could be collected. Sample size was based on previous NF studies using similar study design.^25,26^ Based on previous work in healthy older adults,^25^ the sample size required to detect a NF effect compared to sham [power (1–beta) = 0.80 and alpha = 0.05, independent sample *t*-test] is 18 as calculated using G*Power (version 3.1). Given drop-out and the potentially increased variability in stroke survivors, 30 participants should be sufficient to detect a NF effect.

Twenty-seven (22 males) chronic stroke survivors (>6 months post stroke) met the initial screening criteria and were recruited into the trial. All participants provided written informed consent in accordance with the Declaration of Helsinki and the National Research Ethics service (UK) approved protocol (14/LO/0020). Two participants withdrew from the trial after the initial baseline session and were therefore not randomized (one due to motor cortex lesion, one did not tolerate the MRI environment). One participant’s study participation was halted after the baseline session due to restrictions related to the COVID-19 pandemic. Twenty-four participants (19 males) were randomized following the baseline session (Real = 12, Sham = 12) and completed the NF training. Participants were randomized (1:1 allocation) with minimization of variance in time-since-stroke and baseline Action Research Arm Test (ARAT)^27^ score using a freely available randomization service (www.rando.la). However, the first three participants were forced into the Real group in order to ensure that sham videos were available. Participants were blind to which condition they had been allocated to throughout the experiment. Randomization was performed by a researcher (Z.B.S.), who also set up the NF software to deliver the correct condition but was not involved in any clinical assessments. All clinical assessments were carried out by blinded researchers (T.S., M.M. and M.F.). Blinded researchers also gave all task instructions during NF training and completed debriefing questionnaires.

**Figure 1:**
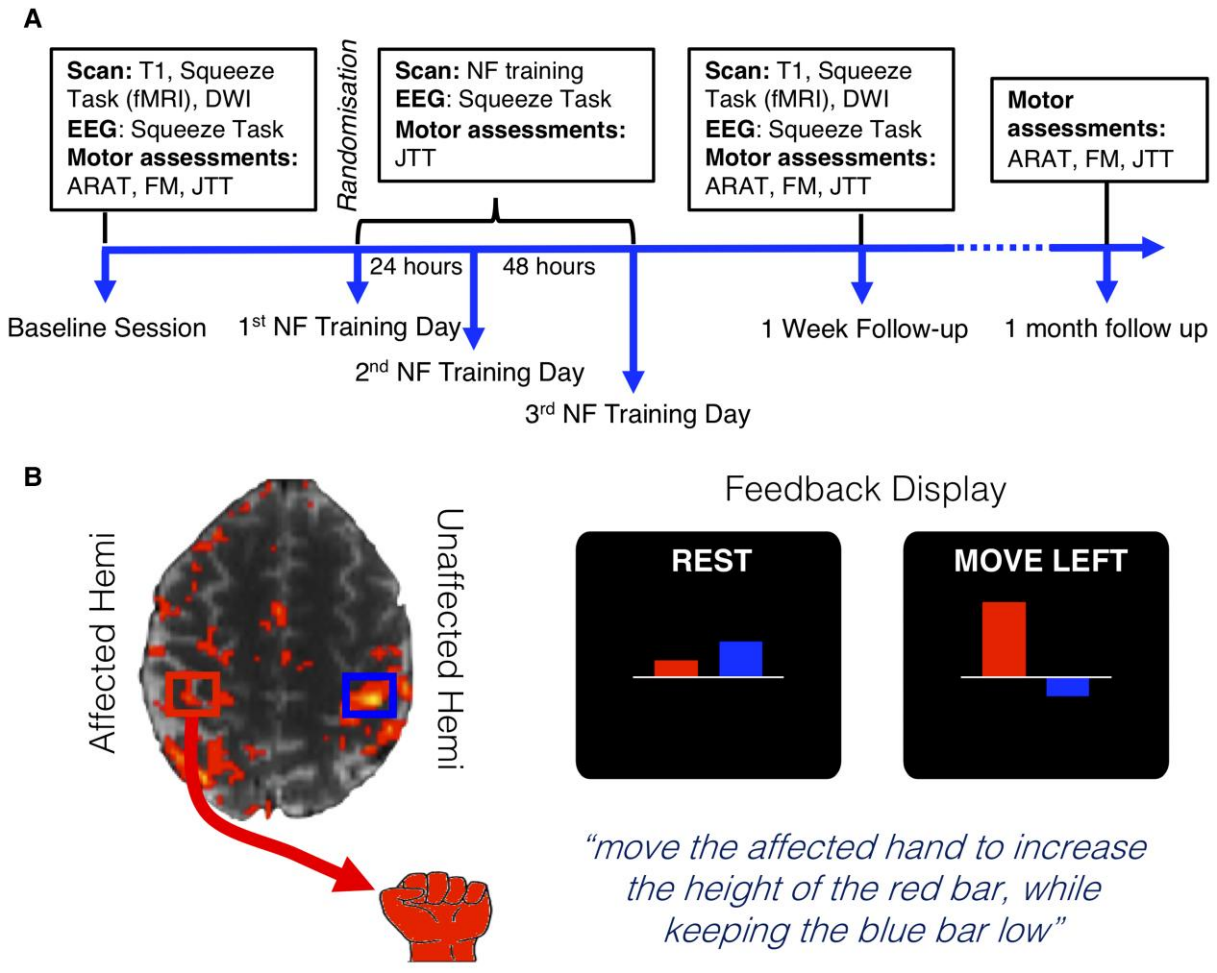
**Study design and timeline.** (**A**) Participants attended three NF training sessions separated by 24 and 48 h, a baseline session and two follow-up sessions at 1 week and 1 month post NF training. (**B**, *Left*) Regions of interest used for NF training were defined during a functional localizer. Regions of interest were centred on peak activity in M1 during movements of both hands. (**B**, *Right*) During NF training, only the stroke-affected hand was moved and participants viewed two bars on the screen. The red bar represented activity in the stroke-affected hemisphere and the blue bar represented activity in the unaffected hemisphere. During movement blocks, participants were instructed to make movements with their stroke-affected hand to increase the size of the red bar, while keeping the blue bar as low as possible.

Participants were invited to attend six testing sessions which took place at the University of Oxford. During an initial baseline session, assessments of upper limb function and impairment were carried out [ARAT, Upper-Extremity Fugl–Meyer assessment (UE-FM), JTT], as well as baseline structural and functional neuroimaging measurements. After the baseline testing day, participants were randomized and attended three NF training sessions, during which they received either Real or Sham NF. Participants then attended two follow-up testing days: one at 1 week post NF where all of the baseline measures were repeated and another 1 month later where only the assessments of upper-limb function were completed (Fig. 1A).

### Neurofeedback intervention

Neurofeedback training was carried out over 3 days, with 24 h between the first two training days and 48 h between the second and third training days. During each NF training day, a functional localizer was initially carried out, during which participants were instructed to open and close their hands at a steady rate that was comfortable for them. There were three 15-s movement blocks for each hand (six in total), interspersed with 15-s rest blocks (total time ∼3.5 min). Participants saw the instructions ‘Squeeze Right’, ‘Squeeze Left’ or ‘Rest’ on the screen, displayed using TurboFeedback (v1.0). A real-time general linear model (GLM) analysis was carried out using Turbo-BrainVoyager (v3.2, Brain Innovation B.V., Maastricht, The Netherlands) and used to identify the peak activity in the hand knob region of the sensorimotor cortex, and two regions-of-interest (1.6 × 1.6 × 1 cm) were centred on the peak activity in each hemisphere (Fig. 1B).

Participants experienced three NF training runs on each day. During NF training runs participants saw two bars on the screen (Fig. 1B), the height of which was determined by the measured percent signal change in the regions of interest (see Supplementary material). Participants were instructed to try different movements with their stroke-affected hand to increase the size of the red bar, while keeping the blue bar as low as possible. This corresponded to increasing activity in the region of interest of the stroke-affected hemisphere (represented by the red bar), while keeping activity in the region of interest of the unaffected hemisphere to a minimum (represented by the blue bar). The height of the bars was continuously updated with each repetition time (TR).

Participants were given some suggested strategies that they could try, including opening and closing their stroke-affected hand or tapping individual fingers (see Supplementary material); however, it was made clear that they could use whatever strategies they wished. Each NF training run consisted of six 30-s NF blocks during which the instruction ‘Move Left’ or ‘Move Right’ (depending on which hand was stroke-affected), was displayed above the bars, and seven interspersed 30-s rest blocks, during which the instruction changed to ‘Rest’. Each NF run lasted ∼6.5 min, totalling 20 min of NF training on each NF training day. Participants were explicitly instructed not to move their stroke-unaffected hand at any point. EMG was acquired to verify this (see Supplementary material). Participants in the Sham group received identical instructions and tasks as participants in the Real group; however, instead of seeing their own brain activity displayed in the NF bars, they saw a video of a previous participant’s feedback.

On each NF training day, before and after the NF training runs, participants completed Pre- and Post-Transfer runs. During these runs, participants were instructed to use movement strategies that they had found to be successful during the NF training runs, when they saw the instructions ‘Move Right’ or ‘Move Left’ (depending on which arm is stroke-affected) on the screen. However, unlike the NF training runs, they did not see the bars on the screen. During the transfer runs, participants received four 30-s movement blocks, which were interspersed with five 30-s rest blocks during which the instructions changed to ‘Rest’. Each transfer run lasted ∼4.5 min. Total scan time was ∼35 min on each NF training day. On the first NF training day, before having experienced any NF training runs, participants were instructed to practice movements with their stroke-affected hand and received some suggested movements to try. After each NF training day, all participants completed a debriefing questionnaire in which they were asked about strategies used during NF and their perceived control over the two bars on a 5-point scale ranging from 1 (not in control) to 5 (fully in control).

### Motor performance measures

The primary outcome measure was performance on the JTT, which was assessed on every testing day. The JTT is a timed assessment that measures performance of fine and gross hand function on six subtasks (handwriting task excluded) that simulate activities of daily living (e.g. simulated feeding). The maximum time permitted for each subtask was 120 s. If the task was not completed in this time, the maximum time was recorded. At baseline three participants could not perform any of the subtasks (Real = 1, Sham = 2); however, these participants improved over time and were able to complete at least two of the subtasks within the time limit. Participants were first familiarized with the task prior to the baseline assessment where they practiced the JTT subtasks until they reached stable performance. Timed data from the JTT was log-transformed to improve normality as the residuals were significantly skewed as assessed by the Shapiro–Wilks test.

Secondary behavioural outcome measures included the ARAT and the UE-FM.^28^ These measures were carried out on the baseline and follow-up testing days to assess changes in stroke-related activity limitation and impairment, respectively.

### Visuomotor squeeze task

Brain function during a controlled visuomotor squeeze task was assessed using fMRI at baseline and 1-week follow-up, and using EEG after each testing session (apart from 1-month follow-up). During the fMRI squeeze task, the participants held an MRI-compatible force transducer (BIOPAC, TSD121B-MRI) in their stroke-affected hand while in the scanner. Before the start of the scan, the participant’s maximum squeeze force was recorded and a target force was determined (∼25% of maximum force). During the scan, participants saw an empty grey bar on the screen. Periodically, a yellow line, which represented their target force, would appear across the grey bar, and participants were instructed to squeeze on the force transducer until a blue bar (which represented the current force exerted on the force transducer) reached the yellow line, at which point they should release. The frequency with which the yellow line appeared (i.e. frequency of squeezing) was set to a comfortable rate for each participant, but was typically between 0.25 and 0.35 Hz. Four 30-s squeezing blocks were interspersed with four 30-s rest blocks during which a fixation cross was presented on the screen.

The EEG visuomotor squeeze task was similar to that described for fMRI. In each session, four movement blocks were performed, with rest breaks between. Each block consisted of ten 5-s trials, with 5.5–6.5 s inter-trial interval. The number of squeezes performed in each trial was set individually for each participant, based on the frequency determined comfortable at baseline (range 1–4 squeezes). EEG data were collected using a 24 channel Ag/AgCl electrode EEG cap (Easycap) and Smarting mobile EEG Amplifier (mBrainTrain LL) following standard scalp preparation techniques (see Supplementary material). Following pre-processing (see Supplementary material), the event related desynchronization (ERD) was calculated for each trial for electrodes C3 and C4 (sensorimotor cortex). For the calculation of the ERD laterality index we used a procedure aligned to the fMRI data analysis, whereby values closer to 1 indicate greater lateralization towards the affected hemisphere.

### MRI acquisition

MRI data were acquired on a 3 T Siemens Magnetom Prisma MRI scanner (Siemens AG) using a 32-channel head coil. All task fMRI was acquired using multiband gradient echo-planer imaging (72 slices, whole brain coverage, voxel-size = 2 × 2 × 2 mm^3^, TR = 933 ms). Additionally, during the baseline and 1-week follow-up scan a whole brain anatomical T_1_-weighted [magnetization prepared rapid gradient echo (MPRAGE), voxel-size = 1 × 1 × 1 mm^3^, TR = 1900 ms, 192 slices] image was collected, as well as DWI using a multishell, echo-planar imaging sequence (voxel-size = 1.75 × 1.75 × 1.75 mm^3^, TR = 2483 ms, 60 diffusion directions, 76 slices). See Supplementary material for full MRI parameters.

### MRI analysis

All fMRI data were pre-processed and co-registered using standard steps within FMRIB Software Library (FSL) (Supplementary material). Data from right-hand-affected participants were mirror-flipped. A first-level GLM was carried out in FEAT for each fMRI run in order to compute task-based statistical parametric maps.^29^ A double gamma-HRF convolved boxcar regressor, as well as its temporal derivative, was used to model the movement and rest blocks, and contrasts were set to Movement > Rest. Group-level analysis was carried out using FMRIB’s Local Analysis of Mixed Effects to compare difference maps between the Real and the Sham groups.^30^ Group *Z*-statistic images were thresholded using clusters determined by *Z* > 3.1 and a family-wise corrected cluster significance threshold of *P* < 0.05 was then applied. The Harvard–Oxford Structural atlases were used to identify the anatomical location of clusters. The probability of the peak voxel belonging to an anatomical structure is reported for each cluster.

DWI data were preprocessed using FMRIB’s Diffusion Toolbox (see Supplementary material). The fractional anisotropy (FA) of the voxels in the corticospinal tract (CST) regions of interest was extracted and any outlier FA values (>3 IQR above median) were removed before averaging. Outlier values were usually restricted to the lesioned area; however, in some participants these were also observed in perilesional tissue, near blood vessels or other abnormal tissues such as cavernomas. CST FA asymmetry was calculated as the difference in mean FA between the two CSTs (Unaffected − Affected)/(Unaffected + Affected), where positive values indicate lower FA/more damage in the stroke-affected CST.

### Laterality and success index calculation

In order to assess changes in laterality of brain activity, a Laterality Index (LI) was calculated based on the magnitude of the fMRI signal change in the regions of interest used for NF training. Laterality indices are traditionally calculated as^31^:

$$LI=\;\frac{ROI_{Aff}-\;ROI_{Unaff}}{ROI_{Aff}+\;ROI_{Unaff}}$$

where ROI_Aff_ and ROI_Unaff_ are fMRI activity in the affected and unaffected regions of interest, respectively. This calculation yields a number between −1 and 1, with positive values indicating lateralization towards the affected hemisphere, and negative values indicating lateralization towards the unaffected hemisphere. As it is possible that the average magnitude of activity in regions of interest (assessed using the average *t*-stat value) is occasionally negative, which results in incorrect or meaningless LI values,^31^ a threshold was applied to the activity in each region of interest using the same approach as in work by Fernández and colleagues.^32^ In brief, for each region of interest on each NF run, the mean maximum activity was calculated as the mean *t*-stat value of the top 5% of voxels showing the highest activity in the region of interest. The threshold of activity that voxels needed to achieve for inclusion in the LI calculation was then set to 50% of this mean maximum activity. This ensured that only activity from the most active voxels was included in the LI calculation and that the average activity for each region of interest was above 0. This approach has previously been validated as more robust and reliable than LI based on unthresholded signal intensity or voxel counts.^33^

A success index was calculated in order to capture variability in NF learning success. As in the current study NF learning appears to occur within (rather than across) NF training days, an NF slope success index was calculated over the runs on each day separately and then averaged. A regression slope was fit across the average LI over each of the NF runs within training days, and then the average slope was calculated over the three training days, resulting in a single NF success index.

### Statistical analysis

For statistical analysis of repeated measures data, linear mixed models (LMMs) were used as implemented in RStudio (version 1.2.5) and the lme4 package.^34^ LMMs allow for incomplete data, as well as greater flexibility in analysis and better handling of repeated-measures dependencies.^35,36^ Random intercepts per participant were included in the random effects structures and the addition of random slopes per participant was assessed using a likelihood ratio test.^37^ There were no cases where the addition of random slopes significantly improved the model fit. *P*-values for fixed effects were derived using the Satterthwaite approximation for degrees of freedom, as implemented in the package lmerTest.^38^ This approach has previously been shown to be less prone to type I errors, and is less sensitive to sample size than other methods.^39^*Post hoc* comparisons were carried out using the emmeans package, with the Tukey method for multiple comparisons. We report unstandardized effect sizes (parameter estimates; *b*) whenever possible in line with general recommendations,^40^ as there is currently no agreed upon way to calculate standard effect sizes for individual terms in LMMs.

For data with just a single follow-up (e.g. FA data) ANCOVAs were carried out comparing the Real and the Sham group at follow-up, while accounting for baseline differences by including baseline as a covariate. Standard effect sizes are reported. The ANCOVA has been shown to be more sensitive in randomized studies compared to ANOVA of change from baseline.^41^

Data that were non-normally distributed were log-transformed where appropriate and/or a sensitivity analysis was performed where any outliers affecting the normality of the distribution were removed and analysis was re-run. In no case did the removal of outliers affect the outcome of the statistical test.

### Data availability

Fully anonymized data from this study can be made available on request.

## Results

Twenty-four participants were randomized into the Real (*n* = 12) and the Sham NF (*n* = 12) groups. Baseline clinical and demographic characteristics are displayed in Table 1. The debriefing questionnaire showed that participants reported using multiple strategies with no clear pattern over days or differences between groups (Supplementary Table 1), and that both groups experienced similar control over the bars (Supplementary Fig. 2). No adverse effects were reported. EMG analysis revealed no significant differences in muscle activity between the Real and Sham group (Supplementary Fig. 3)

**Table 1:** Participant demographics for the Real and Sham groups

Baseline characteristics	Real group (*n* = 12)	Sham group (*n* = 12)
Mean age (range)	63.75 (50–84)	59.25 (36–86)
Male:female	8:4	11:1
Months post-stroke (range)	79.33 (11–230)	70.25 (7–237)
Previous stroke	Y = 1, *N* = 11	Y = 1, *N* = 11
Hand affected	2 Right/10 Left	6 Right/6 Left
Mean JTT (SEM)	267 (75.85)	274 (80.83)
Mean ARAT (SEM)	31.92 (3.63)	34.92 (4.09)
Mean UE-FM^a^ (SEM)	44.04 (2.98)	44.25 (4.18)
Mean FA asymmetry (SEM)	0.12 (0.016)	0.07 (0.018)
Mean Baseline LI (SEM)	0.19 (0.05)	0.22 (0.09)

^a^ UE-FM was scored by two independent blinded assessors and the average score was taken.

### Functional MRI during NF training days

#### Primary outcome: laterality of M1 activity increases with Real NF within days but not across days

**Figure 2:**
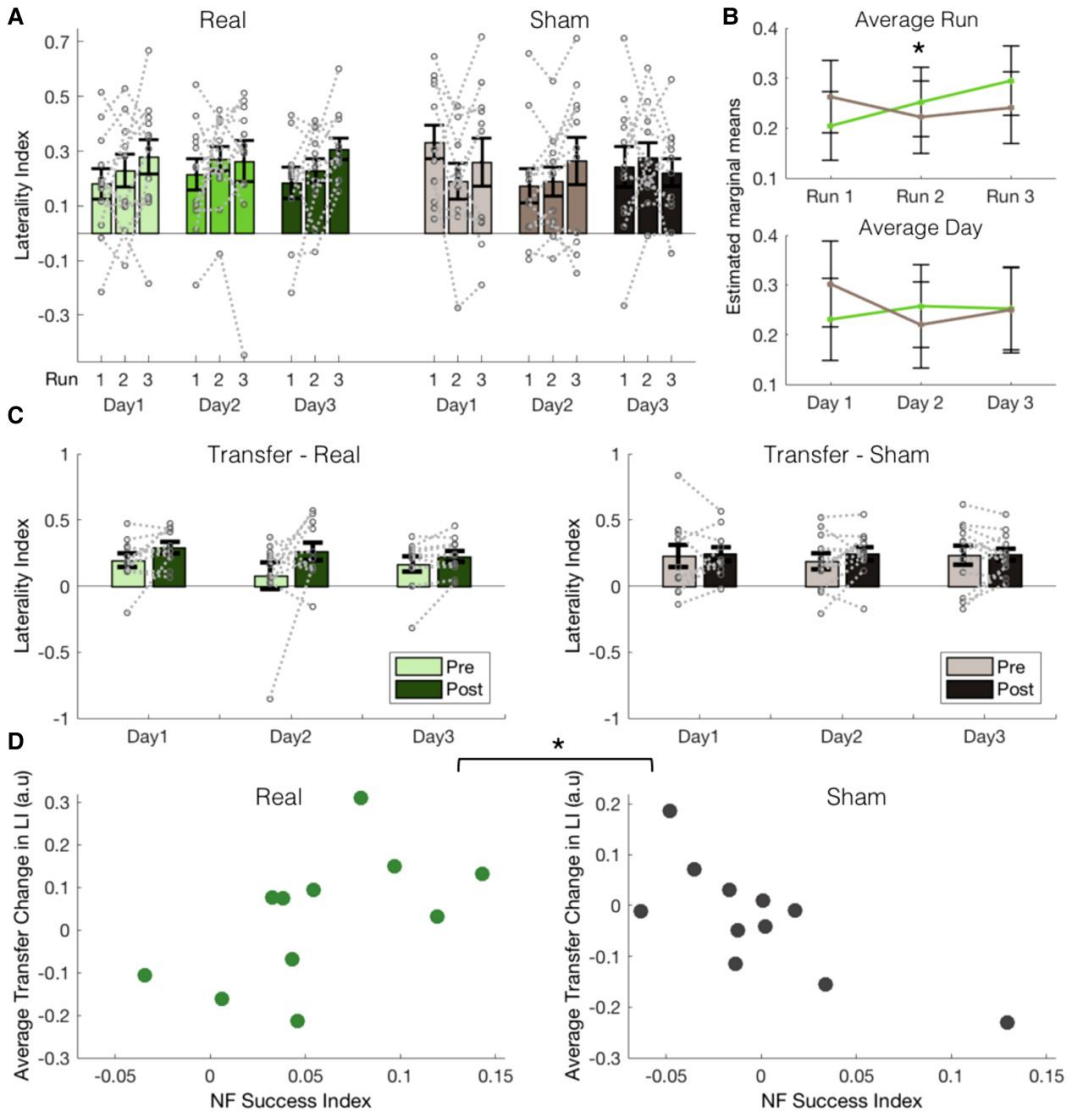
**Laterality of motor cortex activity is increased within, but not across NF training days.** (**A**) Laterality of M1 activity (LI) is displayed for the Real (green/left, *n* = 12) and Sham (grey/right, *n* = 12) group over all the training runs and days. Bars represent group means, grey lines show individual participant data and error bars represent SEM. (**B**, *Top*) When considering NF learning ‘within' training days by averaging LI for each run (pooled over training days), a significant interaction could be observed between Run and Group (*P* = 0.037), with the Real group increasing LI over runs, while the Sham group did not change. Estimated marginal means and confidence intervals are shown for each run. (**B**, *Bottom*) When assessing NF learning *across* training days by averaging LI for each day (pooled over runs), there were no significant interaction or main effects. See also Supplementary Table 4. (**C**) There was no group-level transfer effect; participants in both groups have similar change in LI on the transfer runs (data displayed as in **A**, see also Supplementary Table 5). (**D**) However, the relationship between transfer change and NF success differs between the groups (*P* = 0.002), with a positive relationship found for the Real group, and a negative one for the Sham group. Each dot represents a participant, baseline LI is regressed out of the average transfer change score; therefore, values are centred around zero. For raw values see Supplementary Table 5. There are missing data for one participant in each group due to motion artefacts (see Supplementary material). *P < 0.05.

The first primary outcome measure was the change in laterality of M1 activity during NF training. We hypothesized that the Real group would show increased laterality of brain activity compared to the Sham group. This was assessed first ‘within' training days and then *across* training days. The laterality index of brain activity in the regions of interest used for the NF training was calculated for each NF run on each NF training day (three runs per day, nine in total; see Fig. 2A). Baseline LI was calculated as the LI during the first transfer run and was included as a covariate to correct for any baseline differences in LI.

In order to assess NF learning ‘within*'* training days, the LI for each of the three runs was averaged across all training days for each participant, resulting in average LI values for the first, second and third runs, pooled across training days (Fig. 2B, top). A LMM was fit to the average run LI values, with fixed effects of Group and Run and their interaction, Baseline LI as a covariate and random intercepts per participants. A significant Group × Run interaction was found [*F*(2,46) = 3.556, *P* = 0.04]. After removing one outlier in the Sham group this result remained significant [*F*(2,44) = 3.18, *P* = 0.05]. *Post hoc* pairwise comparisons (using Tukey correction for multiple comparisons; see uncorrected *P*-values in Supplementary Table 2) showed that this interaction was driven by a significant increase in LI between Run 1 and Run 3 in the Real group [*t*(50.4) = 2.815, *P* = 0.019, Run1−Run3: *b* = −0.090, *SE* = 0.032]. No significant differences between runs were found in the Sham group [all: *t*(50.4) < 1.5, *P* > 0.1]. This indicates that the Real NF group were able to increase the laterality of their M1 activity over the runs. Exploration of changes in activity in each hemisphere separately revealed no significant group effect or interaction (Supplementary Table 3). A voxelwise analysis examining differences between average run activity in the whole brain revealed increased activity in the putamen of the unaffected hemisphere in the Real NF group during the first NF runs before decreasing (see Supplementary Fig. 4).

Next, NF learning *across* training days was assessed by averaging LI over runs within each training day for each participant, resulting in average LI values for each day (Fig. 2B, bottom). An LMM was fit to the average day LI values in the same way as described above, but with the fixed effect of Day rather than Run. There was no significant Group × Day interaction [*F*(2,46) = 1.061, *P* = 0.355] or main effect of Group [*F*(1,23) = 0.065, *P* = 0.800] or Day [*F*(2,46) = 0.262, *P* = 0.771], suggesting that LI scores did not change over days and that day-to-day changes in LI did not differ across groups.

#### Secondary outcomes: no evidence for short-term transfer effects following NF training

Secondary fMRI outcomes included exploring participants’ ability to maintain NF learning when feedback was removed. We hypothesized that the Real group would show increased laterality of motor cortex activity after NF training compared to the Sham group. This was assessed during transfer runs before and after NF training on each day. Again, laterality of brain activity during the transfer runs was assessed by calculating an LI based on activity in each of the regions of interest (Fig. 2C). To assess whether there was a within-day transfer effect, a change score was calculated between pre- and post-transfer LI on each day. An LMM was carried out on these data with fixed effects of Group and Day and their interaction, and random intercepts. There was no significant interaction [*F*(2,44) = 0.417, *P* = 0.662] or main effects [Group: *F*(1,23) = 1.642, *P* = 0.213; Day: *F*(2,44) = 2.276, *P* = 0.115]. This suggests that changes in laterality of M1 activity with NF did not persist after the feedback was removed.

High LI variability during NF training and in the transfer runs was observed, with some participants showing large increases in LI, and others showing little or no change in LI (Fig. 2A and C). It was hypothesized that participants who were more successful at using NF would also show the largest increase in LI on the transfer task. In order to quantify how successful individual participants were at using NF, an ‘NF success index’ was calculated as a regression slope over LI of NF runs for each day, and then averaged over training days (see Materials and methods). Where values were missing (see Supplementary material), the slope across the remaining runs was taken.

Average transfer change was calculated as the post–pre difference in LI on the transfer runs per participant (adjusted for baseline LI) averaged across the three training days. The predicted positive correlation was observed in the Real group although it failed to reach significance (Fig. 2D; *n* = 11, Spearman’s *rho* = 0.591, *P* = 0.061, two-tailed), whereas a negative correlation was observed in the Sham group (*n* = 11, *rho* = −0.664, *P* = 0.031, two-tailed). A Fisher’s *Z*-test confirmed that the two correlations were significantly different from each other (*Z* = 2.958, *P* = 0.002).

### Behavioural and clinical measures

#### Primary outcome: improvement on gross motor subtasks following Real NF training

**Figure 3:**
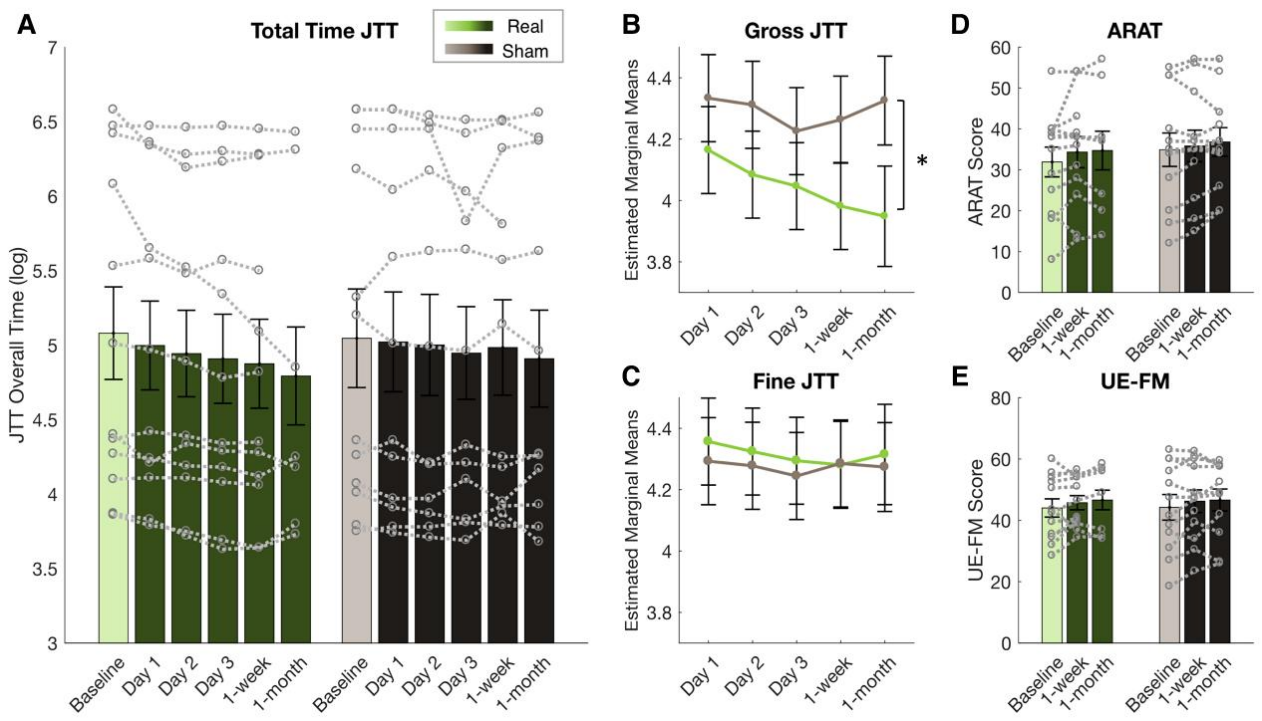
**Participants in Real group improve on gross motor performance (JTT) compared with Sham.** (**A**) Participants in both the Real (green) and the Sham (grey) group got faster at performing the JTT over time when all subtasks were considered (*P* = 0.017) and no group differences were found. Bars represent mean time to complete all subtasks (log), error bars represent SEM and data from individual participants are shown by the grey dotted lines. (**B**) When comparing performance on the gross motor subtasks of the JTT, the Real group had faster performance compared to the Sham group (*P* = 0.01). Estimated marginal means are shown with 95% confidence intervals at each follow-up time point. *P < 0.05. (**C**) The same data are shown for fine motor subtasks of the JTT where no significant group differences were found (see also Supplementary Table 6). There was no group effect on the ARAT (**D**) or the UE-FM (**E**) at 1 week or 1 month after NF training. Data displayed as in (**A**); see also Supplementary Table 7.

The second primary outcome measure of this trial was motor performance changes on the JTT. We hypothesized that the Real group would perform faster than the Sham group. An LMM was carried out on the average time (log-transformed) over all subtasks at each time point (Fig. 3A), with Group and Day as fixed factors, baseline time as a covariate and random intercepts. This revealed a main effect of Time [*F*(4,90.326) = 3.207, *P* = 0.017], with participants in both groups improving over time [*post hoc* linear contrast: *t*(99.3) = −2.818, *P* = 0.006, *b* = −0.205, *SE* = 0.073]. However, there was no main effect of Group [*F*(1,24.225) = 2.660, *P* = 0.116] or Group × Time interaction [*F*(4,90.326) = 1.388, *P* = 0.2445]. The significance of all results was unchanged by removal of one outlier.

In line with previous research,^42–44^ we also separately analysed performance on gross motor subtasks (stacking checkers, moving heavy objects, moving light objects) and fine motor subtasks of the JTT (card turning, picking up small objects, simulated feeding). An LMM was carried out with Group, Day and Task (Gross, Fine) as fixed effects, baseline performance as a covariate and random intercepts. There was a significant main effect of Task [*F*(1168) = 33.374, *P* < 0.001] and Group × Task interaction [*F*(1183.32) = 42.05, *P* < 0.001]. *Post-hoc* pairwise comparisons revealed that this effect was driven by faster performance in the Real group compared to the Sham group on the Gross motor tasks [*t*(34.8) = −2.711, *P* = 0.01; Real–Sham: *b* = −0.247, *SE* = 0.091; Fig. 3B]. In contrast, there was not a significant difference between the two groups on performance of the Fine motor tasks [Fig. 3C; *t*(34.8) = 0.432, *P* = 0.668; Real-Sham: *b* = 0.039, *SE* = 0.091]. While the main effect of Day approached significance [*F*(4156.05) = 2.412, *P* = 0.051], there were no other significant main effects or interactions (all *F* < 2, *P* > 0.1). These findings suggest that participants in the Real group had improved performance on gross motor tasks compared to the Sham group. The significance of all results was unchanged by removal of one outlier.

#### Secondary outcomes: no evidence for Real NF effects on the ARAT or UE-FM

Secondary outcome measures included changes in participants’ performance on two clinical measures, the ARAT and the UE-FM (Fig. 3D and E). We hypothesized that the Real group would have greater improvements on these scores compared to the Sham group. When comparing the two groups at the 1-week follow-up with baseline as a covariate, the two groups were not significantly different from each other on the ARAT [*F*(1,21) = 0.717, *P* = 0.407, $\eta_{p}^{2}$ = 0.033] or the UE-FM [*F*(1,21) = 0.057, *P* = 0.813, $\eta_{p}^{2}$ = 0.003]. Similarly, at the 1-month follow-up there was also no difference between the groups on either measure [ARAT: *F*(1,15) = 0.308, *P* = 0.587, $\eta_{p}^{2}$ = 0.020. UE-FM: *F*(1,15) = 0.055, *P* = 0.818, $\eta_{p}^{2}$ = 0.004]. When all time points were considered in an LMM, both groups improved over time (see Supplementary material).

### White matter structure

#### Secondary outcome: alterations in the corticospinal tract structure after Real NF training

**Figure 4:**
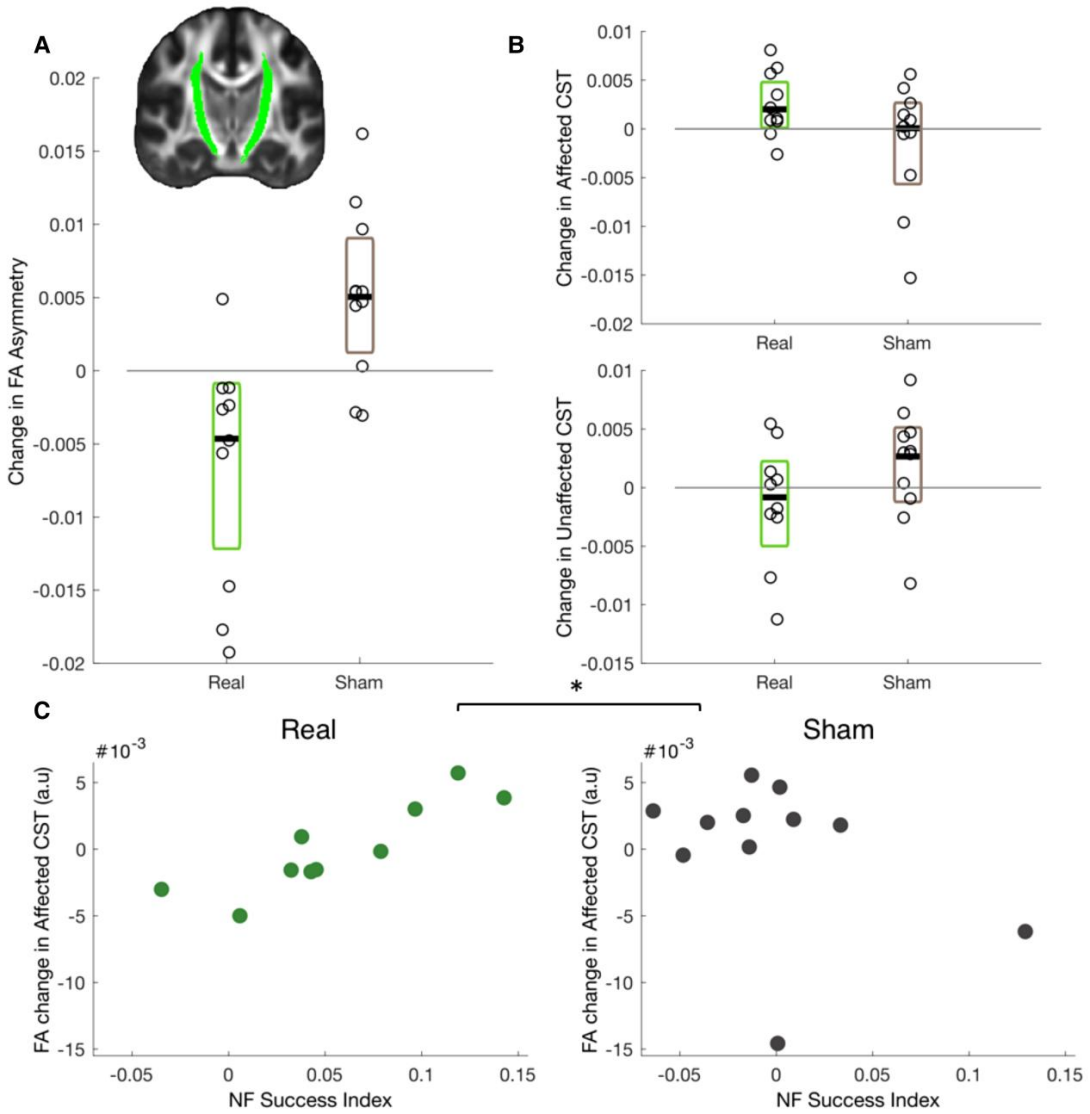
**FA Asymmetry of CSTs is reduced after Real NF training.** (**A**) The Real group (*n* = 10) had lower FA asymmetry in the CSTs (*inset*) after NF training compared to the Sham group (*n* = 11). Change in FA asymmetry between baseline and 1-week follow-up is plotted for the Real and the Sham group. The black line represents median change, coloured boxes represent 95% confidence intervals and individual participant data points are shown with open circles. (**B**) The same information is shown for FA change between baseline and 1-week follow-up in the affected (*top*) and unaffected (*bottom*) CSTs. (See also Supplementary Table 8.) (**C**) Neurofeedback success was positively correlated with FA change in the stroke-affected hemisphere in the Real group, whereas no correlation was found in the Sham group with the two correlations being significantly different from each other (*P* = 0.004). *P < 0.05.

DWI was acquired before and 1 week after NF training. It was hypothesized that the Real group would show greater reduction in FA asymmetry of the CST compared to the Sham group. FA was extracted from the stroke-affected and -unaffected CST and FA asymmetry was calculated (see Materials and methods). FA asymmetry of the CSTs has previously been linked with stroke impairment^45,46^ and was therefore used as the measure of interest here. FA asymmetry decreased (between baseline and 1 week) in the Real NF group and increased in the Sham group (Fig. 4A). An ANCOVA comparing 1-week follow-up values between the two groups, with baseline FA asymmetry as a covariate, revealed a significant group difference [*F*(1,18) = 8.99, *P* = 0.008; $\eta_{p}^{2}$ = 0.333], with the Real group having significantly lower FA asymmetry after NF training than the Sham group (Estimated marginal means: Real = 0.089, Sham = 0.098) when accounting for baseline.

Effects on FA asymmetry can be driven by one or both hemispheres; changes in FA for each hemisphere separately are shown in Fig. 4B. Follow-up ANCOVAs comparing the two groups on each CST separately failed to reach significance [Group effect affected hemi: *F*(1,18) = 2.501, *P* = 0.131, $\eta_{p}^{2}$ = 0.122; unaffected hemi: *F*(1,18) = 1.442, *P* = 0.245, $\eta_{p}^{2}$ = 0.074], but exploratory pairwise comparisons suggest that the change in asymmetry was more likely driven by increases in FA in the affected CST in the Real group [*t*(9) = −2.338, *P* = 0.044, *d* = −0.739], rather than changes in the unaffected CST [*t*(9) = 0.841, *P* = 0.422, *d* = 0.266], or the Sham group [affected: *t*(10) = 0.795, *P* = 0.455, *d* = 0.240; unaffected: *t*(10) = −1.380, *P* = 0.198, *d* = −0.416]. However, the pairwise difference for the affected CST in the Real group would not survive correction for multiple comparisons and should be interpreted with caution. These results were not changed by removal of one outlier in the Sham group.

#### FA change in the affected CST is correlated with NF training success

Additionally, the relationship between FA changes and NF success was also explored. NF success did not correlate with changes in FA asymmetry (adjusted for baseline asymmetry) in the Real group (*rho* = −0.297, *P* = 0.407), or the Sham group (*rho* = −0.164, *P* = 0.657). However, there was a significant correlation between change in FA in the affected CST (adjusted for baseline FA; Fig. 4C) and NF success in the Real group (*rho* = 0.879, *P* = 0.002; two-tailed, Bonferroni corrected alpha for six comparisons = 0.008), but not in the Sham group (*rho* = −0.030, *P* = 0.946, with outliers removed *rho* = 0.12, *P* = 0.78, see Supplementary Fig. 5). A Fisher’s *Z*-test confirmed the two correlations were significantly different from each other (*Z* = 2.622, *P* = 0.004, with outliers removed *P* = 0.012). In contrast, correlations with change in FA in the unaffected CST did not reach significance in either group (Real: *rho* = 0.612, *P* = 0.067, Sham: *rho* = −0.297, *P* = 0.407). This suggests that participants who were more successful at using NF also had the greatest increases in FA in the stroke-affected CST.

### Transfer to visuomotor squeeze task

#### Secondary outcome: no transfer to visuomotor squeeze task

Finally, we used a visuomotor squeeze task (see Materials and methods) to test for any transfer of activity modulation with NF to a constrained task (i.e. same movement parameters pre–post). We hypothesized that the Real group would have increased lateralization of motor cortex activity on this task compared to the Sham group after training.

For the fMRI visuomotor squeeze task, an LI was calculated based on activity in an anatomically defined hand-knob region of interest. A between-subjects ANCOVA on follow-up LI in the two groups, with baseline LI as a covariate, showed no significant differences between the two groups [*F*(1,17) = 0.166, *P* = 0.689, $\eta_{p}^{2}$ = 0.010]. LI at follow-up (adjusted for baseline LI) in the Real group was also not correlated with the NF success index (*rho* = −0.009, *P* = 0.989, *n* = 11, two-tailed). Additionally, no evidence of transfer effects were observed on the EEG visuomotor squeeze task where LI was calculated over all sessions based on the ERD obtained from electrodes C3 and C4 (sensorimotor cortex; see Supplementary Fig. 6).

#### Increased activity in NF learning-related brain regions after training

**Figure 5:**
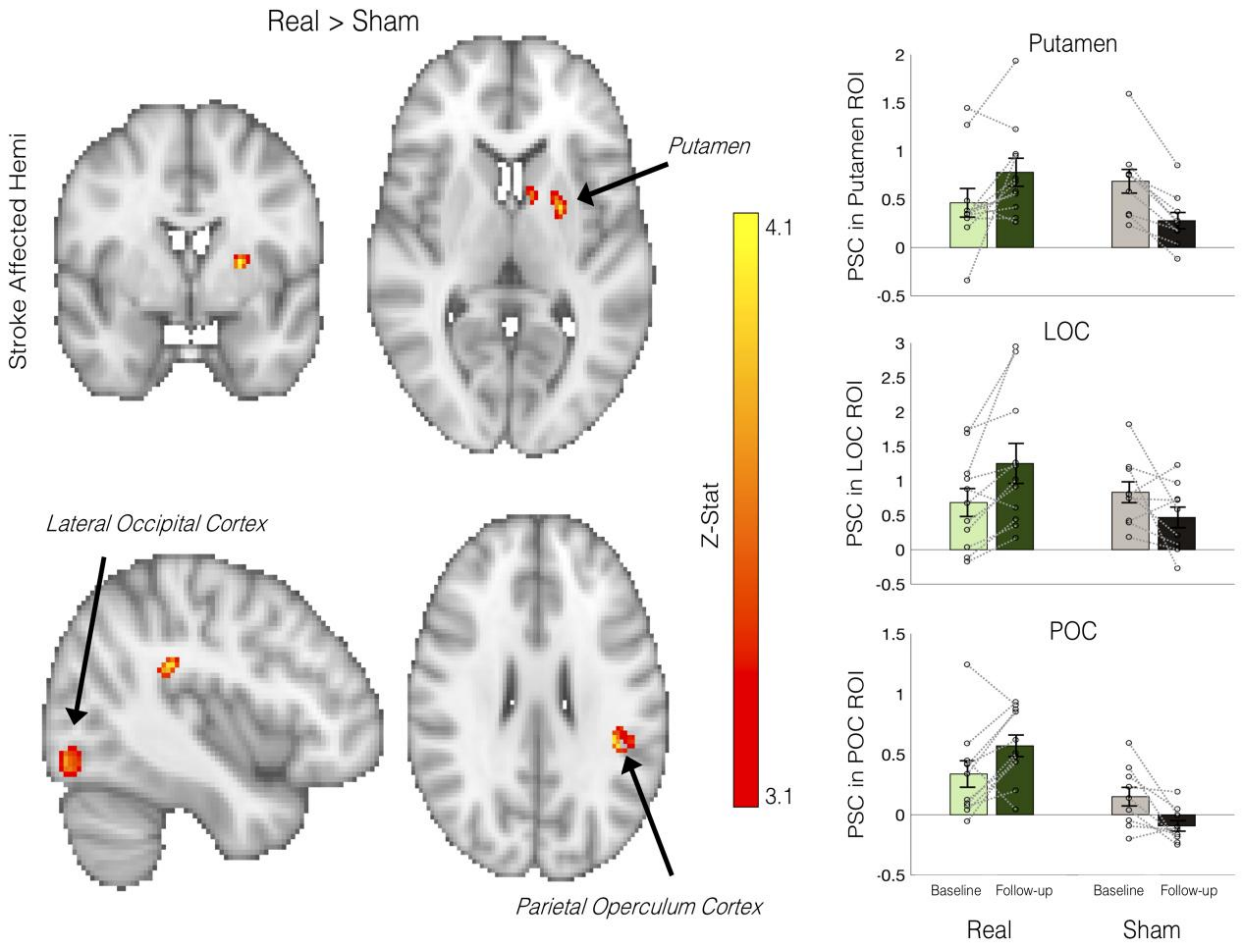
**Increased activity in brain regions associated with NF learning**. Changes in brain activity before and after NF or Sham training were assessed during a controlled visuomotor squeeze task. Three significant clusters were found where the Real group had greater change in activity following NF than the Sham group (Real > Sham, voxelwise GLM, *P* < 0.05, corrected), located in the putamen, LOC and the POC of the unaffected hemisphere. For visualization purposes, the mean percent signal change of the significant clusters is plotted on the right, as well as the data from individual participants (represented by open circles). Error bars represent SEM. See also Supplementary Table 9.

Whole-brain voxelwise analysis comparing the difference between follow-up and baseline brain activity, across the two groups (Real versus Sham) revealed three significant clusters (Fig. 5). Significant clusters were all located in the unaffected hemisphere and included the putamen (*P* = 0.015, MNI coordinate of max zstat: −24 0 8, 99% probability), the lateral occipital cortex (LOC; *P* = 0.041, −38 −82 −14, 52% probability) and the parietal operculum cortex (POC; *P* = 0.047, −42 −38 26, 24% probability). In each cluster the Real group increased activity after NF training, whereas the Sham group decreased or stayed the same. Finally, within the Real group we tested whether baseline brain activity during the squeeze task is associated with subsequent NF performance. While there was no association between laterality of M1 activity at baseline and NF success index (*rho* = −0.336, *P* = 0.290, two-tailed), two significant clusters were found when exploring associations across the whole brain (see Supplementary Fig. 7).

## Discussion

Our study provides the first evidence from a randomized, double-blind, sham-controlled trial on stroke survivors ability to use fMRI NF and its effects on behaviour and the brain. For the primary outcome measures, we found that chronic stroke survivors were able to use fMRI NF to increase laterality of M1 activity during motor execution within NF training days. However, there was no effect of NF training on the primary behavioural outcome measure, average JTT performance. Secondary analysis found improvements in gross hand motor performance (assessed by the gross motor subtasks of the JTT) and alterations in white matter microstructure. This study therefore provides important new evidence on opportunities and challenges for translation of fMRI NF to the setting of stroke rehabilitation.

The finding of successful neuromodulation in the Real group is in line with a series of pilot studies using fMRI^19–21^ or multimodal^47,48^ NF to target a variety of motor regions in stroke survivors, and is a promising indication that NF is a feasible approach in this population. However, one key challenge for any clinical NF intervention is to deliver performance gains that persist after NF is removed. Comparing Real to Sham NF, we did not find NF carry-over effects across training days or transfer effects when the NF display was removed. However, consistent with prior literature,^49,50^ NF training success was highly variable across participants in the Real NF group. There was some indication that this variability in NF success during training may be linked to the persistence of NF gains. Research has begun to investigate factors related to variability in NF learning success,^50,51^ which could allow future studies with larger samples to identify individuals most likely to respond to NF training using baseline measures.

Another key challenge for the development of clinical NF interventions is to drive changes in brain activity that translate into meaningful and long-lasting improvements in clinical symptoms. Here we tested motor function using a range of measures and found that while there was no evidence of improvement on the average JTT performance (the primary behavioural outcome measure), there was evidence of improved performance on the gross motor subtasks of the JTT in the Real group compared to the Sham group. That improvements were found for gross rather than fine subtasks fits with extensive evidence that fine motor tasks are the hardest to recover following damage to the corticospinal system.^52–54^ We did not find any evidence of NF training improving scores on clinical scales of impairment (UE-FM) or activity (ARAT). This lack of an effect on clinical measures may be due to a number of factors. One possibility is that laterality of brain activity may not be a suitable intervention target for all stroke survivors. In particular, it has previously been suggested that more severely affected stroke survivors may instead benefit from boosting activity in both M1s.^5^ Additionally, neuromodulation approaches are often considered not as treatments *per se*, but rather as approaches that enhance the potential for use-dependent plasticity.^55^ Therefore, the addition of further motor rehabilitation outside of the scanner, to drive the motor system while it is ‘primed’, may be necessary to elicit observable effects of NF training on clinical measures. For example, a previous study in Parkinson’s patients found clinically relevant improvements of symptoms after just two sessions of NF combined with extensive practice outside of the scanner using the successful strategies identified during training.^56^ However, the finding of an effect on the gross subtasks of the JTT is a promising first indication that NF can induce behavioural change in this population and ways to bolster this effect should be explored further, especially considering that the current study NF training was conducted on a relatively short timescale (three sessions of 20 min) and without further practice outside the scanner. Previous studies that demonstrated significant motor improvements in chronic stroke survivors are typically conducted over a much longer time period consisting of many hours of training over multiple days or weeks (for example, ∼9 h using Transcranial Direct Current Stimulation and additional motor training,^57^ ∼10 h of EEG NF and additional motor training^58^ or between ∼17 and ∼90 h of motor training).^59,60^

Any long-term benefits of NF are likely to be mediated *via* structural and functional brain plasticity in brain systems engaged in training. To test for structural plasticity, we assessed FA asymmetry in the CSTs, as this measure is consistently found to correlate with motor dysfunction after stroke^61^; therefore, finding ways to strengthen (or rewire) CST output could potentially lead to improved functioning.^62^ NF training led to reduced FA asymmetry and participants who performed better on the NF training exhibited greater increases in CST FA of the affected hemisphere. These results provide the first evidence in a clinical population that fMRI NF can induce structural changes in white matter tracts and supports previous work showing similar effects in healthy individuals targeting motor areas.^26,63^ For example, previous work has shown bi-directional FA changes in the corpus callosum 24 h after NF training.^26^ As FA is sensitive to several white matter features it is difficult to determine the precise neurobiological mechanism underlying changes in FA. While changes in myelination have been shown to occur on this timescale,^64,65^ other biological mechanisms could also underlie the observed effects, such as changes in axon calibre or astrocyte volume.^66^ Recent rodent studies have shown that optogenetically activating the intact contralesional CST after stroke, in combination with intense rehabilitation, led to near-complete recovery of skilled forelimb function as well as corticospinal sprouting from the intact CST to the denervated tract.^67^ However, differences in rodent CST anatomy^68^ as well as intensity of training schedules^69^ may hinder translation of stroke animal models to humans. In humans, while white matter plasticity plays an important role in learning,^66^ there is little evidence of neuromodulation techniques that directly target or boost white matter plasticity. Recent work using Transcranial Magnetic Stimulation NF has demonstrated the feasibility of directly modulating excitability of CSTs in healthy participants^70^; however, it remains to be discovered whether this can lead to behavioural improvements in healthy people or stroke survivors. Our results show that fMRI NF might be a promising tool to target CST structure in stroke survivors, potentially leading to better motor recovery.

In addition to changes in brain structure after NF, we also found functional brain changes during a visuomotor squeeze task. After Real NF training, increased activity in several brain regions that have been implicated in NF learning was observed, including the putamen (dorsal striatum), the LOC and the POC. Currently, the precise mechanism underlying NF learning remains unknown; however, the dorsal striatum has been consistently implicated in NF learning^71–74^ and blocking long-term potentiation in the striatum impairs NF learning.^75^ This has led to the suggestion that NF learning may involve procedural and reinforcement learning.^73,74^ Here we also demonstrated increases in putamen activity during initial NF learning (Supplementary Fig. 4). The finding that activity in the putamen is also increased 1 week after NF training suggests that participants in the Real group may have learnt to engage this area during initial motor performance. Previous research in rodents has shown that connectivity between the dorsal striatum and motor cortex increases with NF training,^75,76^ which may lead to increased activity in the dorsal striatum when the motor cortex is engaged. The LOC has been demonstrated to be a brain region consistently activated during NF learning irrespective of the NF target^72^ and is involved in directing attention to visual signals.^77^ Additionally we found increased activity in the POC. The identification of brain regions that are involved in NF learning helps to improve understanding of mechanisms involved in NF learning, which could help to optimize future studies and could also have implications for individuals who may have damage to these regions.

There were several limitations to this trial. The randomization used in the current study prioritized time since stroke and baseline motor performance; however there were other characteristics that by chance varied between the groups, including gender and which hand was stroke-affected (Table 1). While gender was shown not to be predictive of fMRI NF performance in a recent meta-analysis,^51^ there has been some evidence in the EEG NF field of effects of gender,^78^ and other contextual factors.^79,80^ on NF performance. Additionally, differences in handedness could play a role in maintenance of NF effects,^25^ which should be considered in future studies along with other possible effects on NF learning such as cognitive ability or age. Furthermore, while the sample size in the current study was favourable compared to previous neuromodulation studies in this population, the sample size is still relatively small, meaning that we may have been underpowered to detect some effects and future replication is needed in a larger sample of stroke survivors. This would provide further support for the current findings and would allow more exploration of variability in response. The current findings may, however, provide useful starting points and highlights avenues for further research. Throughout the NF sessions, participants were allowed and encouraged to try different movement strategies. While this limits the amount of experimental control of the movements made, it does allow patients to use NF as a guide to adjust behaviour, by identifying movements that best engage ipsilesional motor cortex which could then be practiced further outside of the scanner.

In order for NF to be a useful rehabilitation tool, clinically meaningful improvements in motor function need to be demonstrated. The addition of motor rehabilitation and more training sessions to potentiate the NF effect could establish whether clinically relevant changes in behaviour can be achieved with fMRI NF. Once this has been demonstrated and effect sizes can be calculated, larger randomized controlled trials can be carried out to account for patient heterogeneity and to compare the effectiveness of this approach to other rehabilitation approaches with similar training schedules, such as with EEG-NF or brain stimulation, and could establish the cost-effectiveness of this approach in comparison to cheaper alternatives. Using fMRI has the advantage of whole brain coverage, allowing monitoring of changes throughout the brain which may shed light on mechanisms involved and off-target changes that may underlie changes in behaviour. However, cheaper and more practical modalities such as EEG would lend themselves to the clinical translation of this approach. fMRI NF is a highly flexible approach and it has become increasingly transparent that it is unlikely there is a one-size-fits-all rehabilitation solution for all stroke survivors, and the need to stratify individuals has been highlighted.^81,82^ Future research could harness the flexibility of this approach to personalize treatments to better suit individual stroke survivors.

## Supplementary Material

awac239_Supplementary_Data

## Abbreviations

ARAT: Action Research Arm Test
CST: corticospinal tract
FA: fractional anisotropy
JTT: Jebsen Taylor hand function Test
LI: Laterality Index
LMM: linear mixed models
M1: primary motor cortex
NF: neurofeedback
UE-FM: Upper-Extremity Fugl–Meyer assessment
